# Lung function in very preterm infants with patent ductus arteriosus under conservative management: an observational study

**DOI:** 10.1186/s12887-015-0480-y

**Published:** 2015-10-24

**Authors:** Hsiu-Lin Chen, Rei-Cheng Yang, Wei-Te Lee, Pei-Lun Lee, Jong-Hau Hsu, Jiunn-Ren Wu, Zen-Kong Dai

**Affiliations:** Department of Respiratory Therapy, College of Medicine, Kaohsiung Medical University, Kaohsiung, Taiwan; School of Medicine, College of Medicine, Kaohsiung Medical University, Kaohsiung, Taiwan; Department of Pediatrics, Kaohsiung Medical University Hospital, No.100 , Tzyou 1st Road, San Ming District Kaohsiung, 807 Taiwan

**Keywords:** Bronchopulmonary dysplasia, Lung function, Patent ductus arteriosus, Very preterm infants

## Abstract

**Background:**

Persistent patent ductus arteriosus (PDA) during hospitalization is thought to be associated with adverse pulmonary outcomes in very preterm infants. This observational study aimed to compare the lung function in very preterm infants with and without PDA at discharge.

**Methods:**

Very preterm infants, admitted to our neonatal intensive unit, who required respiratory support soon after birth and had undergone a lung function test at discharge, were enrolled. Infants with a need for positive-pressure support (either an invasive ventilator, or nasal continuous positive airway pressure without oxygen) or supplemental oxygen at a postmenstrual age of 36 weeks were defined as having bronchopulmonary dysplasia (BPD). Echocardiography was performed weekly for each of the very preterm infants with PDA to confirm closure of the PDA. The data were collected retrospectively.

**Results:**

Fifty-two very preterm infants received lung function tests before discharge during the study period, 28 of whom had PDA and received conservative management, and 20 who did not. The other 4 infants who were given active treatment for PDA were excluded. Gestational age was significantly smaller in the PDA group than in the no-PDA group (27.1 ± 2.0 vs. 28.6 ± 1.6 weeks, *p* = 0.009). Birth weight did not differ significantly in those with and those without PDA (0.98 ± 0.26 vs. 1.12 ± 0.26 kg, *p* = 0.074). Significantly more infants with PDA had BPD (*p* = 0.002) and required respiratory support for a longer period (*p* = 0.001) than those without PDA. However, functional residual capacity (ml/kg) at discharge was comparable between the two groups after adjusting for gestational age and postmenstrual age at testing (21.6 ± 8.4 vs. 21.5 ± 6.7 ml/kg, *p* = 0.894). Other lung function test parameters were also comparable.

**Conclusion:**

Under a definition of BPD (including infants needing CPAP but without oxygen) other than the conventional definition, the very preterm infants in our study who received conservative management for PDA had a higher percentage of BPD than the infants without PDA. The parameters of the lung function test and lung clearance index were comparable between these two groups at discharge.

## Background

Very preterm birth results in neonatal morbidity, primarily due to the functional immaturity of multiple organ systems, such as respiratory distress syndrome, patent ductus arteriosus (PDA), intraventricular hemorrhage, necrotizing enterocolitis, retinopathy of prematurity, and the development of bronchopulmonary dysplasia (BPD) [1, 2].

PDA is a common problem in very preterm infants. Persistent PDA is thought to be associated with adverse outcomes such as necrotizing enterocolitis, intraventricular hemorrhage, BPD, and death in very preterm infants [3, 4]. Large systemic to pulmonary (left to right) ductal shunts may result in an increase in pulmonary circulation, which may lead to pulmonary edema, worsened lung mechanics, decreased lung compliance, and deterioration in gas exchange with hypercapnia and hypoxemia [5, 6]. However, the treatment of persistent PDA in very preterm infants remains controversial [7]. Active management of persistent PDA includes both pharmaceutical and surgical interventions. Because an increasing number of reports have focused on the adverse effects of pharmacotherapy (indomethacin, ibuprofen) [8, 9] and surgical ligation [10], physicians increasingly use conservative management in very preterm infants with PDA.

Currently, in the neonatal intensive care unit of Kaohsiung Medical University Hospital, neonatologists also prefer to treat very preterm infants with PDA conservatively. We hypothesized that persistence of PDA in very preterm infants treated with conservative management may result in worsened lung mechanics and changes in the development of lung function. Therefore, in this observational study, we aimed to compare the morbidity and lung function of very preterm infants with persistent PDA under conservative management and those without PDA at discharge.

## Methods

### Subjects

This was a clinical observational study. Very preterm infants (birth weight <1500 g and gestational age <32 weeks) admitted to the neonatal intensive care unit of Kaohsiung Medical University Hospital in Taiwan who required respiratory support soon after birth and had undergone a lung function test at discharge were enrolled between January 2011 to December 2013. Patients with major birth defects or chromosomal abnormalities were excluded. The clinical data collected included gestational age, birth weight, gender, antenatal use of steroids, severity of respiratory distress syndrome as determined by chest X-ray at admission, postnatal use of exogenous surfactants, and mode and duration of respiratory support. Infants with a need for positive-pressure support (including invasive ventilator, nasal continuous positive airway pressure (CPAP), with any FiO_2_) or supplemental oxygen, at a postmenstrual age (PMA) of 36 weeks were defined as having BPD. We did not use the conventional definition of BPD, i.e., need for oxygen at a PMA of 36 weeks. In our neonatal intensive care unit, cyclic nasal CPAP under room air is the method of choice for weaning from respiratory support, and room air CPAP is stopped when the patients achieve a stable respiratory condition; therefore, patients would not receive only supplemental oxygen in our clinical setting. The conventional definition of BPD is therefore not applicable in such situations.

### Diagnosis of PDA in very preterm infants

In our neonatal intensive care unit, the pediatric cardiologist performs once-weekly echocardiography at the bedside for very preterm infants, and preterm infants in our neonatal intensive care unit are thus echocardiographically assessed once during their first week of life. The echocardiographic criteria for PDA included visualization of the ductus or an increased left atrial to aortic root ratio of more than 1.2, and a left to right shunt of ductal flow seen under color Doppler ultrasound [11–13]. Echocardiography was performed weekly for each of the very preterm infants with PDA to confirm closure of the PDA. The diameter of the duct was also measured and recorded.

The conservative management for the very preterm infants with PDA included fluid restriction (daily fluid up to 130 ml·kg^−1^·day^−1^ beyond day 3), diuretic agents if necessary (furosemide, or trichlormethiazide), appropriate respiratory support (supplemental oxygen, CPAP, or invasive mechanical ventilator), and assessment of symptoms and signs of congestive heart failure. All of the enrolled infants received care according to the established and standard protocols for care of very preterm infants used in our neonatal intensive care unit, including the ventilatory strategy, nutritional policy, infection control, and criteria for blood transfusion.

### Lung function test

The enrolled infants underwent lung function tests once when they were ready for discharge, i.e., when the infants were in a clinically stable condition. The tests were performed using an infant lung function testing system (EXHALYZER® D, ECO MEDICS AG, Duernten, Switzerland), using multiple-breath washout and ultrasonic transit-time measurements [14]. During the examination, pulse rate and oxygen saturation were monitored continuously by a pulse oximeter. Measurements were performed by the same respiratory therapist, using an infant face mask according to the manufacturer's instructions, with the infants in the supine position during unsedated sleep. Functional residual capacity (FRC), tidal volume (TV), respiratory rate, time to peak tidal expiratory flow (T_PTEF_), total expiratory time (T_E_), and indices of ventilation inhomogeneity (lung clearance index, first and second moment ratios) were measured during normal tidal breathing using a tracer gas (4 % SF6) and the multiple-breath washout technique.

### Statistical analysis

The characteristics and parameters of the lung function test of the very preterm infants with and without PDA were compared using the Mann–Whitney U test for numerical data and the chi-square test for categorical data. Multivariate analysis, including gestational age and PMA at testing, was performed to adjust for the gestational differences between the groups. Correlations between the maximum size of PDA and the parameters of the lung function test were assessed using the Pearson correlation test. The required sample size was estimated to be 48 for detecting a difference of 5 ml/kg in FRC between the two groups, with a standard deviation of 6, at an alpha level of 0.05 [15]; thus, 24 individuals would be required per group, which was close to the actual size of the groups. All statistical analyses were performed using JMP® statistical software version 10.0.0 (SAS Institute Inc., Cary, NC). A two-tailed p-value of less than 0.05 was considered to be statistically significant.

### Ethical approval

This study was approved by the Institutional Review Board (IRB) of the Kaohsiung Medical University Hospital, and the IRB number is KMUH-IRB-990282. The informed consent was obtained from all parents.

## Results

**Fig. 1:**
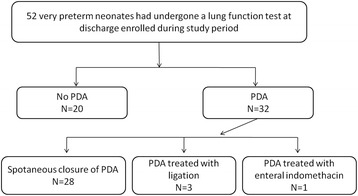
Flow chart of the study infants with data on lung function tests for the study period

Fifty-two very preterm infants underwent lung function tests before discharge between January 2011 and December 2013, 32 of whom had PDA. Three of these infants who were treated with surgical ligation and one who received three doses of enteral indomethacin were excluded. Of the remaining 48 infants, 28 had PDA (the PDA group) and 20 did not (the no-PDA group). All of the 28 very preterm infants with PDA underwent conservative management (Fig. 1). The gestational age was significantly lower in the PDA group than in the no-PDA group (*p* = 0.009). Birth weight was not different significantly between those with and those without PDA (*p* = 0.074; Table 1).

**Table 1:** The clinical characteristics of the study infants

	PDA^a^	No-PDA	*P*-value
	(*n* = 28)	(*n* = 20)	
Birth weight, kg	0.98 ± 0.26	1.12 ± 0.26	0.074
Gestational age, weeks	27.1 ± 2.0	28.6 ± 1.6	0.009
Male, *n* (%)	14 (50)	11 (55)	0.733
Antenatal steroid use, *n* (%)	15 (53.6)	12 (60)	0.658
Intubation after birth, *n* (%)	20 (71.4)	8 (40)	0.029
RDS, Grade 3 or 4, *n* (%)	14 (50)	6 (30)	0.166
Exogenous surfactant use, *n* (%)	8 (28.6)	2 (10)	0.118
Days of invasive ventilation	22.2 ± 28.1	1.6 ± 2.3	0.008
Total days of respiratory support	66.8 ± 25.6	40.8 ± 21.8	0.001
O2 dependence at 28 days, *n* (%)	25 (89.3)	14 (70)	0.092
BPD, *n* (%)	21 (75)	6 (30)	0.002
ROP, ≥ stage 3, *n* (%)	7 (25)	2 (10)	0.189
NEC, *n* (%)	0 (0)	2 (10)	0.087
IVH, any grade, *n* (%)	11 (39.3)	5 (25)	0.301
Pneumothorax, *n* (%)	4 (14.3)	0 (0)	0.136

^a^PDA with conservative treatment

All continuous data are presented as mean ± SD

*RDS* respiratory distress syndrome; *BPD* bronchopulmonary dysplasia; *ROP* retinopathy of prematurity; *NEC* necrotizing enterocolitis; *IVH* intraventricular hemorrhage

The rate of intubation at birth was higher in the PDA group, and the duration of invasive ventilation and respiratory support was longer in the PDA group than in the no-PDA group (Table 1). There were no significant differences in gender, antenatal steroid use, severity of respiratory distress syndrome at admission, use of exogenous surfactants, and complications, including oxygen dependence at 28 days of age, retinopathy of prematurity, necrotizing enterocolitis, and intraventricular hemorrhage, between the two groups. However, 75 % of the infants with PDA had BPD, which was significantly higher than that in the infants without PDA (30 %) (*p* = 0.002).

**Fig. 2:**
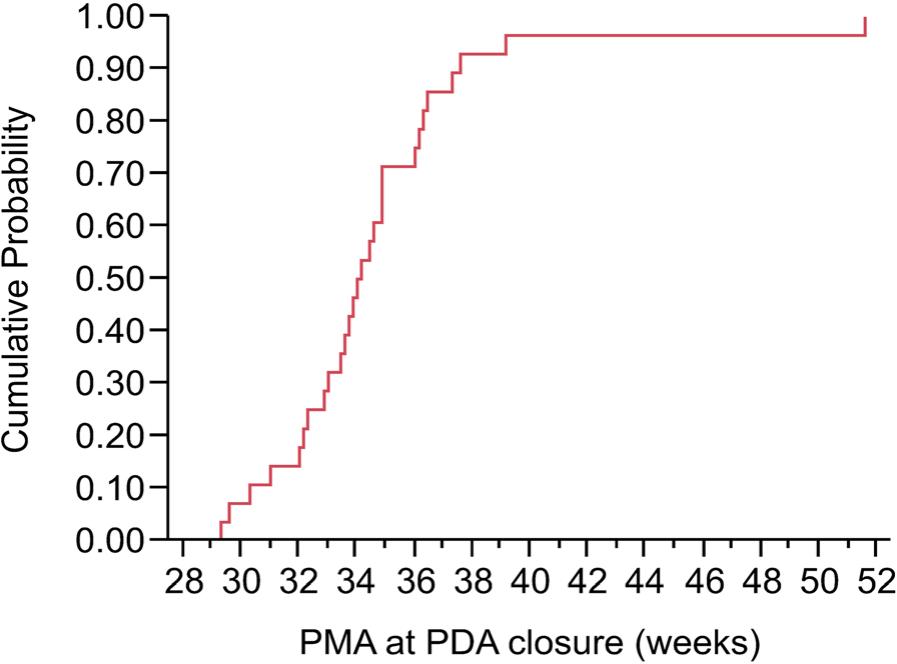
Cumulative distribution function plot showing the cumulative probability of spontaneous closure of PDA at postmenstrual ages (PMA)

Twenty-seven individuals in the PDA group (96.4 %) demonstrated spontaneous closure during hospitalization, but PDA persisted after discharge in the remaining infant. The maximum size of PDA during hospitalization was 3.2 ± 1.1 mm (range: 1.8–4.6 mm) in the PDA group. Spontaneous PDA closure occurred at 50 ± 27.8 days (range: 13–155 days) postnatally, with a mean PMA of 34.6 ± 4.1 weeks (range: 29.3–51.6 weeks). A cumulative distribution function plot showed that the cumulative probability of spontaneous closure of PDA was 0.7 at a PMA of 35 weeks, and more than 0.9 at a PMA of 38 weeks (Fig. 2). All of the enrolled infants were discharged home without oxygen supply or respiratory support.

The results of the lung function tests by multiple-breath washout are shown for both groups in Table 2. Due to the difference in maturity between the two groups when lung function was measured, we further conducted multivariate analysis to adjust for the gestational differences between the groups. After adjusting for gestational age and PMA at testing, there was no statistically significant difference in FRC (ml/kg) at discharge between the two groups (21.6 ± 8.4 ml/kg with PDA vs. 21.5 ± 6.7 ml/kg without PDA, *p* = 0.894). The other parameters of the lung function tests, including TV, respiratory rate, T_PTEF_, T_E_, and indices of ventilation inhomogeneity, were also comparable between both groups after adjustment (Table 2). An adjustment for PMA alone or postnatal age was also performed, and again there were no significant differences in FRC or other parameters of the lung function tests between the two groups (data not shown).

**Table 2:** Comparison of pulmonary function parameters in the two groups

	PDA^a^	No-PDA	*P* value
	(*n* = 28)	(*n* = 20)	
Postnatal age at testing, days	91.8 ± 30.7	69.2 ± 23.1	0.012^b^
PMA at testing, weeks	40.4 ± 2.8	38.8 ± 3.0	0.063^b^
Body weight at testing, kg	2.71 ± 0.58	2.65 ± 0.59	0.385^b^
Body length at testing, cm	46.7 ± 4.9	46.5 ± 4.1	0.690^b^
FRC (ml)	57.1 ± 19.5	56.4 ± 19.0	0.580^c^
FRC (ml/kg)	21.6 ± 8.4	21.5 ± 6.7	0.894^c^
FRC (ml/cm)	1.23 ± 0.44	1.20 ± 0.33	0.866^c^
Vt (ml)	23.3 ± 4.9	23.4 ± 4.6	0.430^c^
Vt (ml/kg)	8.8 ± 2.4	9.0 ± 1.5	0.432^c^
MV (ml/kg/min)	1359.9 ± 693.5	1358.3 ± 536.0	0.778^c^
RR (/min)	78.1 ± 20.2	80.2 ± 15.0	0.637^c^
T_I_ (s)	0.39 ± 0.10	0.36 ± 0.08	0.151^c^
T_PTEF_ (s)	0.16 ± 0.06	0.20 ± 0.15	0.390^c^
T_PTEF_/T_E_ (%)	41.1 ± 16.4	46.0 ± 18.4	0.759^c^
LCI	15.4 ± 4.4	16.2 ± 3.4	0.745^c^
M1/M0	5.0 ± 2.2	5.1 ± 1.7	0.529^c^
M2/M0	41.1 ± 16.4	46.0 ± 18.4	0.887^c^

^a^PDA with conservative treatment

^b^compared using the Mann–Whitney *U*-test

^c^ *P* value: after adjustment with GA, and PMA at testing

All continuous data are presented as mean ± SD

*FRC* functional residual capacity; *Vt* tidal volume; *T*_*I*_ inspiratory time; *T*_*PTEF*_ time to peak tidal expiratory flow; *T*_*PTEF*_ */T*_*E*_ the ratio of time to peak tidal expiratory flow over total expiratory time; *LCI* lung clearance index; *M1/M0* and *M2/M0*, first and second moment ratios

Because detrimental effects are thought to be directly related to the magnitude of the PDA shunt, we further performed correlation analysis between the maximum size of the PDA and parameters of the lung function tests in the PDA group, but found no significant correlations.

## Discussion

Our results showed that lung volume and indices of ventilation inhomogeneity in very preterm infants receiving conservative management for PDA were comparable to those of very preterm infants without PDA at discharge, although the infants with PDA had a higher percentage of BPD than those without PDA.

PDA is a common condition in very preterm infants; however, the management of PDA in very preterm infants is still a controversial issue. Bose and Laughon stated that the patency of the ductus arteriosus may be a physiological phenomenon in very preterm infants [16]. The EPICure study [17] reported that approximately 65 % of preterm infants born at a gestational age of less than 28 weeks who survive to discharge have persistent PDA. Regarding the natural course and rate of spontaneous closure of ductus arteriosus in very preterm infants, previous studies [18, 19] reported a spontaneous PDA closure rate of 66–94 % after conservative management in very preterm infants born at a gestational age of less than 30 weeks. In this observational study, the spontaneous closure rate of PDA in the infants receiving conservative management was 96.4 %, which was compatible with the high rate of spontaneous closure of PDA in very preterm infants. We also found that there is a direct relationship between PMA and the spontaneous closure of PDA in very preterm infants (Fig. 2). Thus, maturity is important for spontaneous closure of ductus arteriosus.

Persistent ductal patency is thought to be associated with adverse outcomes, such as hemodynamic derangements and several major complications, including necrotizing enterocolitis, intraventricular hemorrhage, BPD, and death in premature infants [3, 4]. With concerns over adverse effects of aggressive treatment of PDA, conservative management is increasingly considered to be an option [18]. Jhaveri et al. reported that the rates of BPD, retinopathy of prematurity, neurological injury, and death are similar between infants receiving conservative management and those undergoing early PDA ligation [20]. In this study, we showed there were no significant differences in major complications between infants with PDA who underwent conservative management and those without PDA. However, the rate of BPD (including infants needing CPAP, but without oxygen, at a PMA of 36 weeks) was higher in the infants with PDA than in those without PDA (75 % vs. 30 %, *p* = 0.002). Since, in our neonatal intensive care unit, we apply room air CPAP (i.e., oxygen equals 21 % when using CPAP) for most very preterm infants with acceptable oxygen saturation (88–94 %) [21], the definition we use for BPD is different from the diagnostic criteria developed by the National Institute of Child Health and Human Development Workshop on BPD in 2001 [22], in which BPD is defined as treatment with oxygen of more than 21 % at a PMA of 36 weeks, plus a total oxygen duration of more than 28 days for infants born at less than 32 weeks’ gestation. The use of oxygen therapy as a single criterion for defining BPD has limitations in clinical situations [5]. We considered that lung function is impaired when infants require positive-pressure ventilation or CPAP, even though oxygen requirement equals 21 % at a PMA of 36 weeks.

In this study, we were concerned about whether pulmonary sequelae differed between very preterm infants with and those without PDA. With PDA, large systemic to pulmonary (left to right) ductal shunts result in an increase in pulmonary flow and pulmonary remodeling in preterm infants. As a result, these infants need prolonged respiratory support to maintain adequate ventilation, which then increases the likelihood of respiratory morbidity [6, 23]. Bancalari et al. reported a strong association between the presence and duration of PDA, and an increased risk of developing BPD in premature infants [6]. However, the association between PDA and BPD may be distorted by confounding factors, such as gestational age and other co-morbidities [24]. A recent study by Laughon et al. reported that the role of PDA as an independent risk factor for BPD was less marked [25]. Therefore, the lung functions in very preterm infants with persistent PDA under conservative management should be addressed when they are ready to be discharged.

FRC is frequently used for monitoring lung disease during infancy, and FRC has been found to be reduced in infants with BPD in some studies [26–29]. However, we found that FRC was not lower in the patients with PDA compared to those without, although there was a higher rate of BPD in infants with PDA under conservative management. Furthermore, some authors have suggested that normalized FRC in infants with BPD may result from both hyperinflation and/or gas trapping due to airway obstruction [30]. The amount of time to reach peak tidal expiratory flow to total expiratory time (T_PTEF_/T_E_ ratio), which is recognized to be a tool for evaluating airway obstructions [31], has been reported to be lower in infants with airway obstruction and lower compliance [32, 33]. Our patients may not have had airway obstructions severe enough to reveal a significant difference in the T_PTEF_/T_E_ ratio between those with and without PDA, because there was no difference in T_PTEF_/T_E_ ratio between the two groups.

Increased lung clearance index and moment ratio values imply reduced ventilation efficiency in infants [28, 34, 35]. We did not find any differences in lung clearance index or moment ratio values between the PDA and no-PDA groups, suggesting that the very preterm infants with prolonged PDA did not have greater ventilation inhomogeneity than those without PDA.

The major limitation of this study was that the lung function tests used can detect changes in volume-dependent pulmonary mechanics and airway function, but not specific changes in the lung periphery. New BPD infants are characterized by an arrest in lung development, with simplification of the lung periphery. The simplification of the lung periphery includes arrested alveolar growth, with fewer, larger, and simplified alveoli, and fewer and dysmorphic arteries [36]. In future, methods for evaluating changes in the lung periphery, such as volumetric capnography [36], should be considered. The other limitations of this observational study are that we did not include infants with PDA under active management, due to the small number of such patients during the study period, and because the sample size overall was small. The potential strength of this study is that we examined lung function, after the PDA had closed spontaneously, to determine whether there were residual effects on lung function due to its earlier occurrence.

## Conclusions

In our study, very preterm infants who received conservative management for PDA had a higher percentage of BPD, defined as infants needing CPAP, but without oxygen, than infants without PDA. The parameters of the lung function test and lung clearance index were comparable between these two groups at discharge. Long-term follow up of pulmonary outcomes in later life is necessary.

## Abbreviations

BPD: bronchopulmonary dysplasia
PMA: postmenstrual age
CPAP: continuous positive airway pressure
FRC: functional residual capacity
IVH: intraventricular hemorrhage
LCI: lung clearance index
M1/M0 and M2/M0: first and second moment ratios
NEC: necrotizing enterocolitis
RDS: respiratory distress syndrome
ROP: retinopathy of prematurity
T_I_: inspiratory time
T_PTEF_: time to peak tidal expiratory flow
T_PTEF_/T_E_: the ratio of time to peak tidal expiratory flow over total expiratory time
Vt: tidal volume
